# Amyloid Structural Changes Studied by Infrared Microspectroscopy in Bigenic Cellular Models of Alzheimer’s Disease

**DOI:** 10.3390/ijms22073430

**Published:** 2021-03-26

**Authors:** Agnes Paulus, Anders Engdahl, Yiyi Yang, Antonio Boza-Serrano, Sara Bachiller, Laura Torres-Garcia, Alexander Svanbergsson, Megg G. Garcia, Gunnar K. Gouras, Jia-Yi Li, Tomas Deierborg, Oxana Klementieva

**Affiliations:** 1 Medical Microspectroscopy Laboratory, Department of Experimental Medical Science, Lund University, 22184 Lund, Sweden; agnieszka_agnes.paulus@med.lu.se (A.P.); anders.engdahl@med.lu.se (A.E.); 2 Experimental Neuroinflammation Laboratory, Department of Experimental Medical Science, Lund University, 22184 Lund, Sweden; yiyi.yang@med.lu.se (Y.Y.); antonio.boza_serrano@med.lu.se (A.B.-S.); sara.bachiller@med.lu.se (S.B.); megg.garcia@med.lu.se (M.G.G.); 3 Experimental Dementia Research Unit, Department of Experimental Medical Science, Lund University, 22184 Lund, Sweden; laura.torres-garcia@med.lu.se (L.T.-G.); gunnar.gouras@med.lu.se (G.K.G.); 4 Neural Plasticity and Repair Unit, Department of Experimental Medical Science, Lund University, 22184 Lund, Sweden; alexander.svanbergsson@med.lu.se (A.S.); jia-yi.li@med.lu.se (J.-Y.L.); 5 Lund Institute for Advanced Neutron and X-ray Science (LINXS), 22370 Lund, Sweden

**Keywords:** FTIR, amyloid-β, Tau, α-synuclein β-sheet, cellular environment, Alzheimer’s disease

## Abstract

Alzheimer’s disease affects millions of lives worldwide. This terminal disease is characterized by the formation of amyloid aggregates, so-called amyloid oligomers. These oligomers are composed of β-sheet structures, which are believed to be neurotoxic. However, the actual secondary structure that contributes most to neurotoxicity remains unknown. This lack of knowledge is due to the challenging nature of characterizing the secondary structure of amyloids in cells. To overcome this and investigate the molecular changes in proteins directly in cells, we used synchrotron-based infrared microspectroscopy, a label-free and non-destructive technique available for in situ molecular imaging, to detect structural changes in proteins and lipids. Specifically, we evaluated the formation of β-sheet structures in different monogenic and bigenic cellular models of Alzheimer’s disease that we generated for this study. We report on the possibility to discern different amyloid signatures directly in cells using infrared microspectroscopy and demonstrate that bigenic (amyloid-β, α-synuclein) and (amyloid-β, Tau) neuron-like cells display changes in β-sheet load. Altogether, our findings support the notion that different molecular mechanisms of amyloid aggregation, as opposed to a common mechanism, are triggered by the specific cellular environment and, therefore, that various mechanisms lead to the development of Alzheimer’s disease.

## 1. Introduction

Despite extensive, decades-long research on Alzheimer’s disease (AD), there is still no effective treatment to prevent or stop this neurodegenerative disorder. AD is typically characterized by a build-up of aggregated proteins, including amyloid-β (Aβ), Tau, and alpha-synuclein (αSyn), that have a complex interactions in the brain with, for example, lipids [1,2,3,4]. The majority of AD cases typically involve more than two neuropathologies, [5] and the co-existence of aggregated Aβ, αSyn, and Tau has been associated with more rapid cognitive decline [6,7,8,9,10]. The widely accepted amyloid hypothesis [9,10,11,12] posits that Aβ is a critical trigger of AD [11]. Therefore, the development of anti-amyloid drugs that target Aβ plaques to reduce Aβ load has been the focus. However, the failure of clinical trials targeting Aβ [12], although recent reports of an immunotherapy using aducanumab have been encouraging [13], indicates that the mechanisms of Aβ neurotoxicity are more complex than previously thought [14], and further research is needed to understand the specific role of Aβ and its various molecular species in the pathogenesis of AD. Powerful imaging techniques, such as magnetic resonance imaging and positron emission tomography, have been successfully used to detect the presence of fibrillary amyloids and, in some cases, of oligomeric species extracellularly [15,16,17], other methods are needed to detect intracellular amyloid and characterize the structure of these aggregates [18].

It has been suggested that amyloid proteins such as Aβ and αSyn can form oligomers—supramolecular structures consisting of several noncovalent assemblies [19] and characterized by structural diversity [20] due to the partial enrichment of β-sheet structures, which may define the neurotoxicity of oligomers [21]. Still, the relative contribution of amyloid-specific secondary structures to neurotoxicity remains unknown. Conformation-specific antibodies [22] can detect amyloid aggregates but do not help with characterizing the β-sheet structural content, which may vary between tissues [23,24] and cells [25]. Here, we evaluate the β-sheet structural content of amyloids in biological samples using Fourier Transform Infrared microspectroscopy (μ-FTIR) [26,27,28]. There are three main advantages of using FTIR to study the molecular structure of amyloids: (1) μ-FTIR is sensitive enough to detect β-sheets [29]. (2) μ-FTIR is label-free, and (3) it is a non-destructive technique. μ-FTIR detects infrared absorbance in the mid-IR region (4000–1000 cm^−1^), corresponding to changes in the vibrational energy state of functional groups characteristic of biological molecules. Importantly, for μ-FTIR, labeling is not required; therefore, all non-volatile structural components that could be affected by or lost during labeling remain in situ and contribute to the resulting infrared spectrum [30]. μ-FTIR is a sensitive technique that allows spectra acquisition from sample quantities as low as 0.1 ng [31]. Additionally, it is important to mention that infrared energy is too low (0.05–0.5 eV) to break chemical bonds [32]. Here, we analyzed absorbance intensities corresponding to the bands Amide I (1800–1600 cm^−1^) and Amide II (1570–1470 cm^−1^) in the infrared spectrum to characterize the main components of amyloids and to reveal the secondary structure of proteins via carbonyl (C=O) stretching vibrations in the peptide bonds, allowing for the specific detection of β-sheets and random coil structures [26].

The absorption associated with the Amide I band originates from stretching vibrations of the C=O of the amide group. The infrared absorption associated with the Amide II band originates from bending vibrations of N–H bonds. Early empirical frequency-structure studies found that β-sheets have absorption bands at 1640–1610 cm^−1^ and 1695 –1680 cm^−1^; random coil and α-helix structures are located in the ranges of 1650–1637 and 1660–1650 cm^−1^ [33,34,35,36,37].

More recent studies have found that the principal components of amyloid-β aggregates can be detected as absorbance at 1628 cm^−1^ and that the aggregates are characterized by intra- and inter-molecular β-sheet structural content (1985 and 1691 cm^−1^) [37,38]. It has also been suggested that amyloid oligomers can be partially unordered (increased content of random coils). Thus, the absorbance peak at 1637 cm^−1^ can be used for further protein structure characterization as a ratio between the unordered structure band at 1637 cm^−1^ and Amide I (1656 cm^−1^) [39]. As a measure of protein folding, we used the Amide II/Amide I ratio; to measure cell damage, we monitored for lipid oxidation, defined as absorption at 1740 cm^−1^ by the carbonyl group of the ester bond in the infrared spectrum [40].

This work aimed to study the structural changes of amyloid aggregation in cultured neuron-like mouse cells stably overexpressing human amyloid precursor protein (APP) with the Swedish mutation (N2aAPP_SWE_) [41], this mutation is commonly used in models of AD because it strongly enhances overall Aβ production [42,43]. To understand better interaction of Aβ, αSyn and Tau within a cell, we produced bigenic cells overexpressing (1) APP_SWE_ and αSyn, (2) APP_SWE_ and Tau. Using immunofluorecence labeling we assessed fibrilization of Aβ; using infrared spectroscopy we characterized structural changes directly in neuron-like cells simultaneously expressing human Aβ and αSyn, Aβ and Tau. Obtaining new information about AD-related structural changes in proteins is an important step in understanding the molecular mechanisms behind AD; these results could also provide insight into the pathology of other amyloidogenic diseases.

## 2. Results

### 2.1. αSyn and Tau Affect the Quality of Intracellular Aβ Aggregates

**Figure 1:**
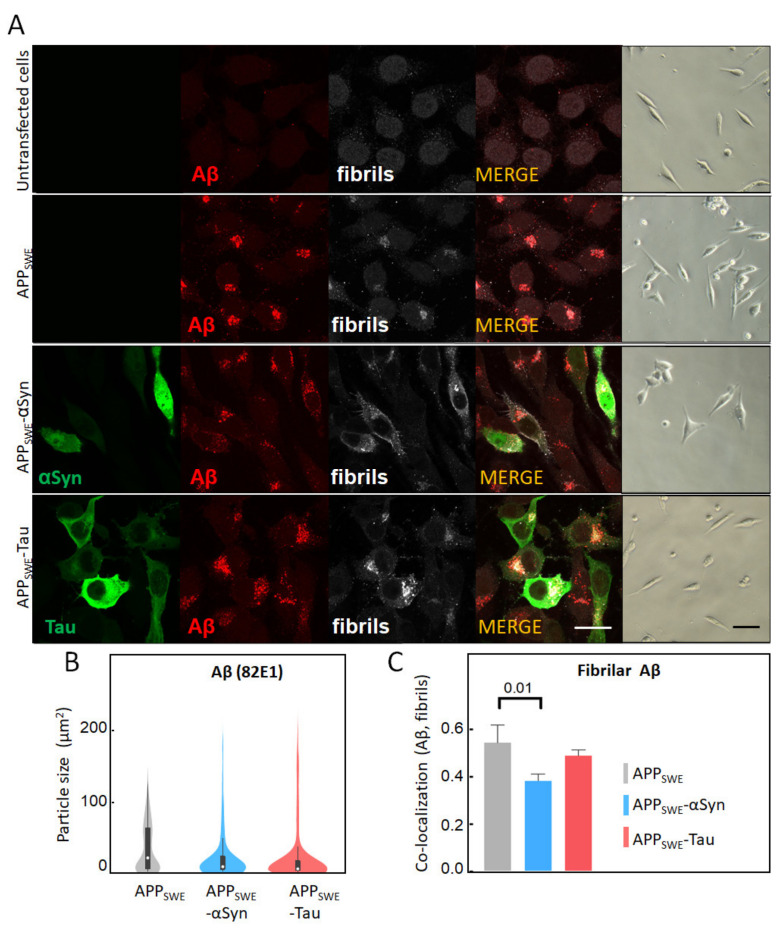
Immunofluorescent characterization of Aβ in cellular models of Alzheimer’s disease (AD). (**A**) Immunofluorescent labeling of Aβ with 82E1 (red) and amyloid fibrils with OC78 (white). The green fluorescence channel corresponds to mutated Tau (P301L) or αSyn (A53T) coupled to GFP. Bright-field images show the condition of fluorescence-activated cell sorting (FACS)-sorted cells (Supplementary Figures S2–S5). The scale bar is 20 µm. (**B**) Quantification of particle size was calculated using surface areas in immunosignal corresponding to 82E1 immunoreactivity. Aggregated Aβ was computed by co-localization analysis of Aβ (82E1) and amyloid fibrils (OC78). A threshold was set at 5% of max size (Supplementary Figure S6). (**C**) Statistical analysis of the co-localization of Aβ with fibrillary Aβ: one-way ANOVA (*p* < 0.01) ± s.d; *n* = 25–35 cells per sample. A coefficient of 1 is equal to 100% co-localization.

To study AD protein aggregation, we used a mouse neuroblastoma (N2a) cell line that was stably transfected with human APP harboring the Swedish mutation (N2a-APP_SWE_). N2a-APP_SWE_ cells overexpress Aβ(1–42), which can then form intracellular β-sheet rich aggregates [41]. To study the effect of human αSyn and Tau on Aβ aggregation, we generated additional cellular models, wherein human αSyn or Tau were expressed together with APP_SWE_. To that end, N2a-APP_SWE_ cells were transfected with plasmids encoding for either αSyn or Tau coupled to GFP (Supplementary Figure S1). In all cell models, Aβ expression was analyzed by immunolabeling, using the Aβ-specific antibody 82E1 [44] and the amyloid fibril-specific antibody OC78 [45] (Figure 1). We did not observe any significant changes in Aβ expression levels between cell lines (Figure 1A); however, an analysis of the signal distribution showed a two-fold decrease in the size of Aβ aggregates in the presence of Tau or αSyn (Figure 1B). Our results also revealed that the Aβ co-aggregation level (OC and 82E1), calculated as co-localization with Aβ-αSyn or Aβ-Tau using the Pearson’s coefficient, was significantly decreased (22%) in the presence of co-expressed αSyn, in N2a-APP_SWE_-αSyn cells (*p =* 0.016) (Figure 1C). These results indicate that αSyn’s presence affects the structure and morphology of Aβ aggregates.

### 2.2. αSyn Reduces β-Sheet Load in a Bigenic Aβ/αSyn Cellular Model

To further study protein aggregation in the presence of co-expressed human Aβ, αSyn, and Tau, we used synchrotron-based Fourier Transform Infrared microspectroscopy (µFTIR). Historically, µFTIR cell studies have been conducted using synchrotron radiation sources due to high brightness that allow the use of a smaller aperture for the synchrotron measurement (6 µm^2^ compared to the 20 µm^2^ available in the laboratory sources) [30,46]. Using µFTIR we studied subtle structural changes directly in the individual cells grown on the µFTIR sample support surface. Specifically, we monitored wavenumbers corresponding to several characteristics of interest: (1) major component characteristics for β-sheet structures (1628 cm^−1^) together with a minor component of the β-strands (1691 cm^−1^); (2) random coils (1637 cm^−1^); and (3) the relative intensity of Amide II (1557 cm^−1^) (Table 1 and Table 2).

**Figure 2:**
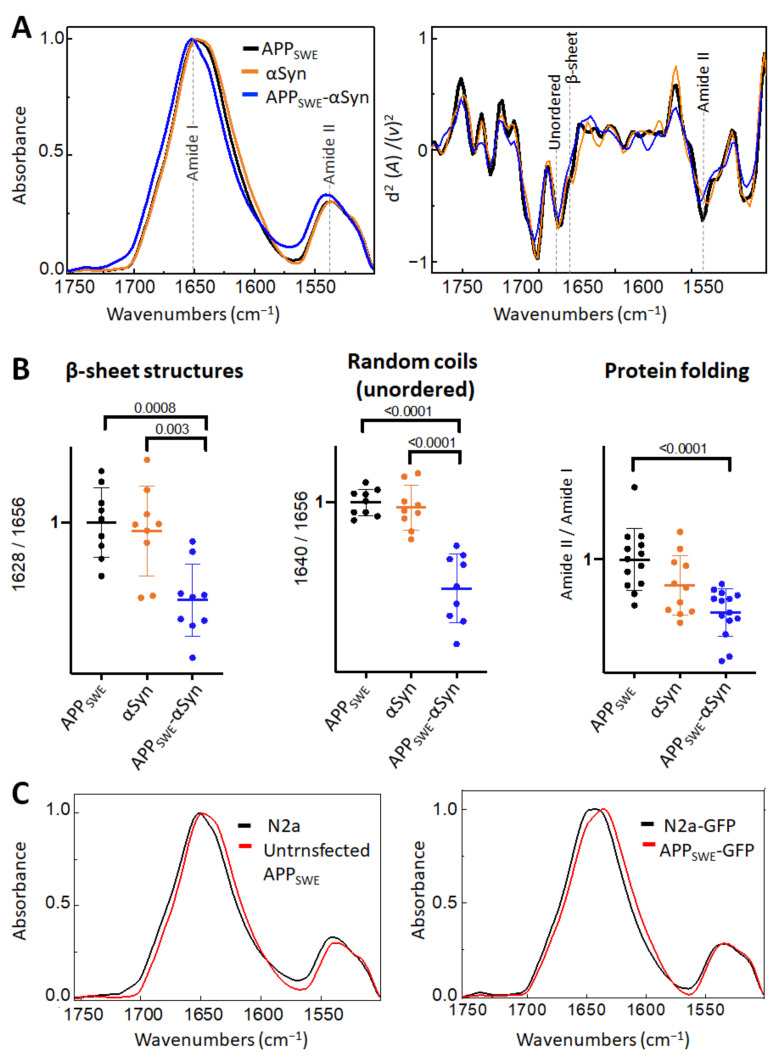
Effect of αSyn (A53T) on Aβ aggregation in Aβ/αSyn cellular model of AD. (**A**) Averaged and normalized infrared absorbance spectra and corresponding normalized second derivatives of N2a-APP_SWE_, N2a-αSyn, N2a-APP_SWE_-αSyn. Dashed lines indicate peak positions. (**B**) Statistical analysis of structural changes. One-way ANOVA (*p* < 0.01) ± s.d; one dot corresponds to a single cell; *n* = 7–20 cells per independent measurement reproduced at least twice. All values are normalized to the control—N2a-APP_SWE_ cells. (**C**) Averaged and normalized infrared absorbance spectra of N2a-APP_SWE_ and N2a cells untransfected (**left panel**) and transfected with GFP (**right panel**).

First, we studied the effect of mutated αSyn (A53T) on Aβ aggregation (Figure 2). The A53T mutation promotes the formation of αSyn fibrils in vitro, leading to a Parkinson’s disease-like pathology [47]. Our data showed that the presence of Aβ and αSyn in N2a- APP_SWE_-αSyn cells led to a significantly reduced level of β-sheet structures compared to in monogenic N2a-APP_SWE_ cells and N2a cells expressing human αSyn (N2a-αSyn). This decrease was accompanied by a significant reduction in random coils (unordered) structures and changes in total protein folding (Figure 2B), indicating a possible reduction in aggregate toxicity via reduced protein folding. We observed an effect of GFP on β-sheet structures (Figure 2C); therefore, to consider the contribution of GFP in the structural analysis, N2a-APP_SWE_ cells transfected with GFP only (APP_SWE_) were used as a control.

### 2.3. Aβ Reduces the Level of β-Sheet Aggregates in the Bigenic Aβ/Tau Cellular Model

**Figure 3:**
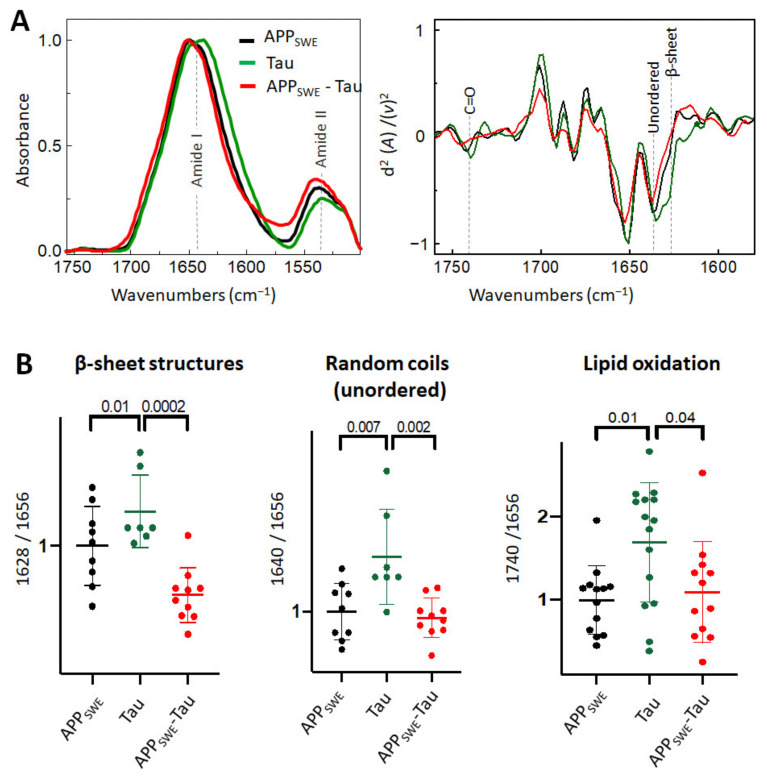
Effect of Tau (P301L) on Aβ aggregation in Aβ/Tau cellular model of AD. (**A**) Averaged and normalized infrared absorbance spectra and corresponding normalized second derivatives of N2a-APP_SWE_, N2a-Tau, N2a-APP_SWE_-Tau. Dashed lines indicate peak positions (**B**) Statistical analysis of structural changes. One-way ANOVA (*p* < 0.01) ± s.d; one dot corresponds to a single cell, *n* = 7–20 cells per independent measurement, reproduced at least twice. All values were normalized to the control—N2a-APPSWE cells.

As a next step, we studied Aβ aggregation in the presence of Tau containing the P301L mutation (Figure 3). Immunofluorescence data (Figure 1C) combined with FTIR analysis showed that the presence of Tau reduced the average size of Aβ aggregates without a significant change to the β-sheet structural load (Figure 3B). Interestingly, in N2a cells expressing Tau alone (N2a-Tau), the levels of β-sheet load, random coils structures, and lipid oxidation were significantly increased compared to Aβ-expressing cells (N2a-APP_SWE_ and N2a-APP_SWE_-Tau cells).

### 2.4. Diverging Patterns of Amyloid Aggregation in Monogenic and Bigenic Cellular Models

**Figure 4:**
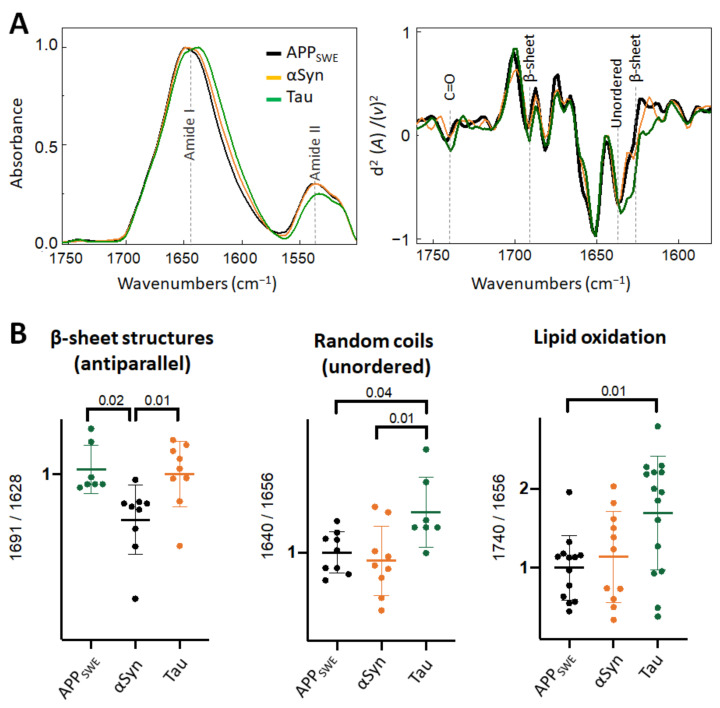
Comparison of structural changes in protein aggregates in the monogenic models of protein aggregation related to AD. (**A**) Averaged and normalized infrared absorbance spectra and corresponding normalized second derivatives of N2a-APP_SWE_, N2a-αSyn, N2a-Tau. Numbers in parenthesis indicate corresponding graphs with statistical analysis. Dashed lines indicate peak positions. (**B**) Statistical analysis of structural changes: one-way ANOVA (*p* < 0.01) ± s.d. All values were normalized to the control, N2a-APP_SWE_ cells. One dot corresponds to a single cell, *n* = 7–20 cells per independent measurement reproduced at least twice.

When comparing monogenic cellular models of amyloid aggregation (Figure 4), we observed that the anti-parallel β-sheet load was significantly lower in cells that expressed Aβ compared to αSyn or Tau only. In N2aAPP_SWE_-Tau, we documented a decreased level of Amide II absorbance (Figure 4A) and increased level of lipid oxidation (Figure 4B), that was significantly different from cells expressing only Aβ.

**Figure 5:**
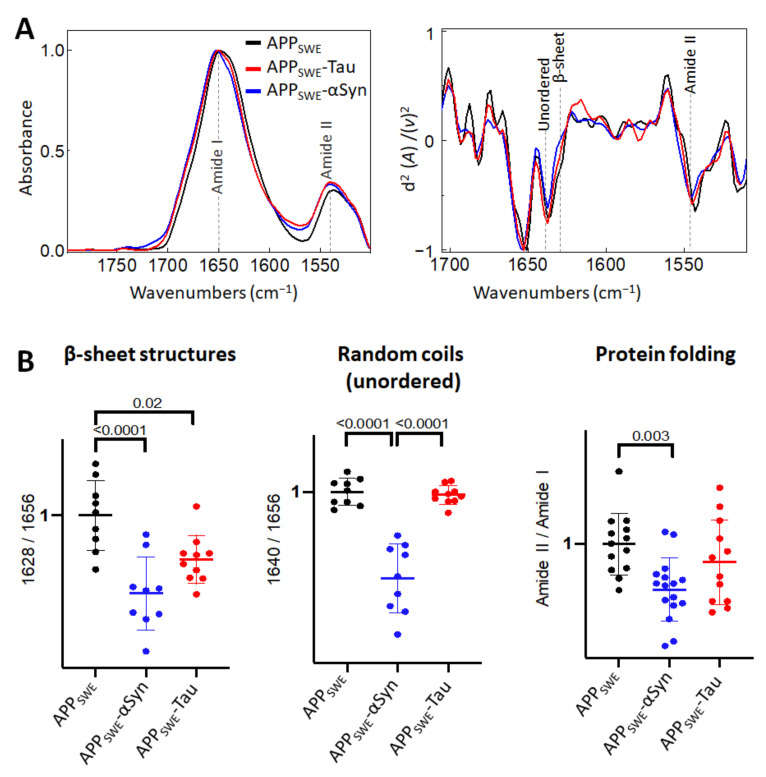
Comparison of structural changes in protein aggregates in the bigenic models of AD protein aggregation. (**A**) Averaged and normalized infrared absorbance spectra and corresponding normalized second derivatives of N2a-APP_SWE_, N2a-APP_SWE_-αSyn, and N2a-APP_SWE_-Tau, models of mixed protein aggregation. Numbers in parentheses indicate corresponding graphs with statistical analysis. Dashed lines indicate peak positions. (**B**) Statistical analysis of structural changes: one-way ANOVA (*p* < 0.01) ± s.d. All values were normalized to the control, N2a-APP_SWE_ cells. One dot corresponds to a single cell, *n* = 7–20 cells per independent measurement reproduced at least twice.

In the bigenic cellular models of amyloid aggregation (Figure 5), we observed that the total β-sheet load was significantly decreased, in contrast to the total β-sheet load in cells that expressed only Aβ. This implies that both αSyn and Tau can affect Aβ aggregation.

## 3. Discussion

Based on our results, we speculate that in human neurons, wherein all three proteins are physiologically present, the formation of amyloid aggregates may involve different folding, aggregation, and post-translational modification processes depending on the cellular environment and pathological conditions. 

There is still no consensus on the neurotoxicity of aggregated Aβ, αSyn, and Tau, which hinders progression when targeting these proteins for therapeutic intervention. To understand the molecular mechanisms behind the toxicity of amyloids, it is necessary to elucidate the relationship between structure and neurotoxicity. However, characterizing the structure of these amyloid aggregates is extremely challenging primarily because of their high heterogeneity. Thus, identifying their structure, which may hint at their neurotoxicity, is fundamental in identifying new therapeutic targets. Studies on amyloid aggregates, so-called amyloid oligomers, typically rely on brain tissue processed by various techniques, including homogenization, high-speed centrifugation, and SDS (sodium dodecyl sulfate) gel electrophoresis. However, such procedures can alter protein conformations or states of assembly. Hence, no consensus exists regarding amyloid aggregate structures in the brain, their association with AD pathogenesis, and their respective contribution to the pathogenesis [14,48].

In this study, we used synchrotron-based µFTIR [32] to measure β-sheet structural content, protein folding [49,50], and lipid oxidation in order to determine amyloid toxicity [30,51]. µFTIR provides simultaneous measurements over the entire wavenumber range, producing broad information about molecular structures [36,37]. Since all molecules are continuously vibrating, almost all molecular bonds absorb infrared energy, and this absorption is measurable by µFTIR spectroscopy in situ [36]. The label-free and non-destructive properties of µFTIR were crucial for this work due to the high fragility and heterogeneity of amyloid structures in cells [25]. 

Aggregated Aβ, αSyn, and Tau have been detected in postmortem brain tissue of AD patients, raising the possibility that these amyloids could interact and generate toxic aggregates [3,52,53]. We examined Aβ aggregation in our monogenic and bigenic cellular models of amyloid aggregation. To understand better interaction of Aβ, αSyn and Tau within a cell, we used neuron-like mouse cells N2a stably overexpressing human APP with the Swedish mutation (APP_SWE_) [41]. This mutation is immediately adjacent to the β-secretase site in amyloid precursor protein, resulting in the substitution of two amino acids: lysine and methionine to asparagine and leucine, respectively, that strongly enhances overall Aβ production [42,43]. Historically, µFTIR cell studies have been conducted using synchrotron radiation sources due to high brightness of the synchrotron beam [30,51]. Using high brightness of synchrotron sources, it is possible to spectra of analytical grade quality from individual cells which is challenging to achieve by commercially available FTIR microscopes. However, with the recent development of optical photothermal spectroscopy [54], a new benchtop solution is available to study protein structures [25]. Here, we used synchrotron-based µFTIR to study molecular structures in neuron-like cells stably in Aβ/αSyn and Aβ/Tau bigenic cells. Thus we produced a cell that co-express (1) Aβ and αSyn with aggregation-prone A53T mutation [52], (2) Aβ and Tau containing the P301L mutation, which promotes neurofibrillary tangle formation [53].

Thus, our findings provide novel evidence that αSyn and Aβ can modulate the structure of β-sheet aggregates in cellular models. Our study demonstrated that bigenic APP/αSyn neuron-like cells display reduced β-sheet load and an altered oligomeric Aβ profile. On the other hand, bigenic APP/Tau neuron-like cells display reduced β-sheet load compared to the monogenic Tau model and an altered oligomeric Aβ profile. 

In addition to the uncertainties surrounding the interactions between amyloid oligomers, the contribution of αSyn and Tau to changes in the amyloid load itself remains unresolved in AD [3]. Additionally, the exact relationship between changes in amyloid oligomer structures and the cellular environment has not been clearly defined [55]. In our immunofluorescence analysis, we unexpectedly noticed that αSyn and Tau affect both the size and quality of intracellular Aβ aggregates. Combining these results with the µFTIR data, we observed that expression of αSyn (A53T) in N2a-APP_SWE_ neuron-like cells resulted in a decreased β-sheet load and that the effect was bidirectional. In contrast, the presence of aggregation-prone Tau did not affect β-sheet load in N2a-APP_SWE_ cells. Our data are supported by in vivo experiments that showed markedly reduced amyloid deposition in bigenic animals that overexpress human wild-type αSyn and Aβ [56]. Taken together, our data show that bigenetic models that involve αSyn and Tau in conjunction with Aβ can offer new functional insight into AD development. The current models of amyloid aggregation used in studies represent rare conditions, wherein two mutated proteins are expressed in the cells; however, our combination of monogenic and bigenic models allows one to study bidirectional effects on amyloid protein aggregation. In this study, we looked at amyloid proteins that are known to be physiologically present in human neurons and explored how they can interact and alter each other. Such structural interactions are important to study as they may lead to the formation of more neurotoxic amyloid aggregates. It is also possible that the neurotoxicity seen in AD can be attributed to other factors, such as altered cellular structures or cells that affect neural cells (i.e., microglia are known to be crucial in AD pathogenesis) [57]. Considering the complex effects of the interaction between αSyn, Aβ, and Tau on their aggregation, further development of µFTIR is required for fluorescence-guided measurements of the β-sheet load in different cellular environments. Further studies will help better understand the relationship between amyloid aggregate structure and toxicity. In future studies, it will be important to include other cellular readouts to detect neurotoxicity, including altered electrophysiological properties, such as gamma oscillations [58], and direct/indirect actions by the glial population [59].

In conclusion, our findings suggest that αSyn and Tau contribute to structural changes in Aβ in AD. Furthermore, our study demonstrates that bigenic APP/αSyn and APP/Tau neuron-like cells display changes in β-sheet loads along with altered oligomeric Aβ profiles. Thus, our findings support the notion that there are a multitude of molecular mechanisms driving amyloid aggregation (depending on the particular cellular environment and conditions), which will be important to consider when studying and treating proteinopathic neurodegenerative diseases.

## 4. Materials and Methods

### 4.1. Plasmids

αSyn: The lentiviral plasmid encoding αSyn (A53T)-GFP was produced by Gibson assembly using the 2nd generation lentiviral plasmid pHsCXW as recipient and a previously generated fusion construct of αSyn (A53T)-GFP as a donor. Briefly, the backbone was linearized by restriction enzymes, SgfI (#FD2094, ThermoFischer Scientific, Gothenburg, Sweden) and MluI (#FD0564, ThermoFischer Scientific, Gothenburg, Sweden), and the αSyn (A53T)-GFP fusion sequence was amplified using the following primers:

Primer 1: cgttagacgcgatatggatgtattcatgaaaggac; Primer 2: cgagacgcgtgtcacttgtacagctcatccatgc. 

Assembly of the construct was performed through one-pot assembly using NEB 2x Gibson assembly master mix (New England Biolabs, NEB-E2611L, Ipswich, MA, USA), following the manufacturer’s protocol.

Tau: Similarly, the lentiviral plasmids containing Tau-GFP (P301L) were generated by Gibson Assembly. The pHsCXW backbone was linearized by enzymatic restriction with BamHI (#FD0054, ThermoFisher Scientific, Gothenburg, Sweden) and SalI (#FD0644,ThermoFisher Scientific, Gothenburg, Sweden). Tau and GFP sequences were amplified from previously generated constructs with the following primers for Tau:

Primer 3: catagaagacaccgactctagagatggctgagccccgccag; Primer 4: caccaccactaccacccaaaccctgcttggccagg) and with the following primers for GFP amplification: 

Primer 5: gcagggtttgggtggtagtggtggtggtagtggtggtatggtgagcaagggcgag; 

Primer 6: tagggcgctcgagacgcgtgttacttgtacagctcgtccatg).

Assembly of the fragments was done using NEB 2x Gibson Assembly Master Mix (#NEB-E2611L, New England Biolabs, Ipswich, MA, USA) following the manufacturer’s instructions.

### 4.2. Cell Lines

We used a mouse neuroblastoma cell line (N2a) stably transfected with human amyloid precursor protein harboring the Swedish mutation (APP_SWE_), which overexpresses Aβ and can form intracellular, β-sheet-rich aggregates [60]. To study the effect of αSyn and Tau on Aβ aggregation in the N2a-APP_SWE_ cell line, we transfected cells with either αSyn (A53T)-GFP and Tau (P301L)-GFP plasmids. Cells were plated in 10-cm Petri dishes and grown in conditioned media containing 47% high glucose Dulbecco’s modified Eagle’s medium (DMEM) (# SH30243.01, GE Healthcare Life Sciences, Uppsala, Sweden), 47% Optimem (#31985-047, ThermoFisher Scientific, Gothenburg, Sweden), 5% fetal bovine serum (FBS) (# 10500-064, ThermoFisher Scientific, Gothenburg, Sweden), and 1% penicillin/streptomycin at 37 °C in a humid 5% CO_2_ incubator. N2a-APP_SWE_ cells were grown in the same medium in the presence of with 0.8% geneticin (#10131-027, ThermoFisher Scientific, Gothenburg, Sweden). 

At 70–80% cell confluence, cells were transfected with plasmids and Lipofectamine 2000 (#11668-027, ThermoFisher Scientific, Gothenburg, Sweden) according to the manufacturer’s instructions. Controls used in the study (N2a-APP_SWE_ and N2A) were also treated with Lipofectamine 2000 (ThermoFisher Scientific, Gothenburg, Sweden). Fluorescence-activated cell sorting (FACS) was used to select GFP-transfected cells with plasmids containing either aSyn, Tau, or GFP only. 24 h after transfection, the cells were trypsinized using Trypsin-EDTA^TM^ solution (#15303651, ThermoFisher Scientific, Gothenburg, Sweden), centrifuged for 1 min at 1000× *g*, and resuspended in phosphate-buffered saline (PBS) (#10010-015, Thermo Fisher Scientific, Gothenburg, Sweden). Later, the single-cell suspensions were incubated with a viability dye, Draq7^TM^ (#424001, Biolegend, San Diego, CA, USA), at a dilution of 1:1000 for 10 min at room temperature in darkness. Thereafter, cells were sorted by a BD FACS Aria III flow cytometer (Santa Cruz Biotechnology, Solna, Sweden) and plated in a 24-well plate containing conditioned media (50% conditioned and 50% new media) at a density of 100,000 cells per well. Data were analyzed using FlowJo^TM^ software (Becton, Dickinson, and Company, Ashland, MA, USA). All cell lines used in the study were treated using the same protocol and conditions. 

### 4.3. Synchrotron-Based FTIR Microspectroscopy 

S-μFTIR was performed at the SMIS beamline at the synchrotron SOLEIL (L’Orme des Merisiers, 91192 Gif Sur Yvette Cedex, France). For microspectroscopy, the FACS-sorted cells were seeded onto clean 1-mm CaF_2_ windows (Crystran Ltd., Dorset, UK) and left to adhere on the surface. After two days of growth, cells were fixed in 4% paraformaldehyde (PFA) solution (Solveco, Rosersberg, Sweden) for 20 min at room temperature, washed 3 × 10 min in PBS, and then rinsed for 10 min in sterile water.

Single point microscopy was performed using a ThermoFisher Scientific Continuum XL FTIR microscope with a 32× magnification, 0.65 NA Schwarzschild objective. FTIR spectra were acquired in transition mode with a spectral range between 4000 and 1000 cm^−1^ at 4 cm^−1^ spectral resolution with 8 µm × 8 µm aperture dimensions using 256 co-added scans. Background spectra were collected from a clean area of the same CaF_2_ window. All measurements were made at room temperature. 

### 4.4. Data Analysis

Orange software (University of Ljubljana, Slovenia) was used to analyze FTIR spectra after atmospheric compensation using OPUS software (Bruker, Ettlingen, Germany). Derivation of the spectra to the second-order using the Savitzky-Golay method with third polynomial order and seven smoothing points was used to unmask the number of discriminative features and eliminate the baseline contribution. Spectra exhibiting high noise and spectra with strong Mie scattering were eliminated. After baseline correction, all the spectra were normalized. Based on several studies, a consensus has been reached on the assignment of IR components in the Amide I region: 1630−1615 cm^−1^ is ascribed to the main intermolecular β-sheet content and the 1697−1690 cm^−1^ region is ascribed to β-sheet (weak). The 1655−1638 cm^−1^ region is defined by random coils, and the 1660−1656 cm^−1^ region indicates α helices [36,37], as summarized in Table 1. To analyze protein structures, we used the spectra ratios [40], as summarized in Table 2. A band centered around 1740 cm^−1^ is mainly assigned to stretching vibration in the carbonyl group of the ester bond ν(C=O) in lipids (triglycerides) [61,62,63]. As a sign of oxidative stress, we used 1740/1656 ratio [40]. Due to the complex nature of the sample, we only used the relative comparison between samples.

### 4.5. Aβ Immunofluorescent Labeling and Confocal Imaging

Cells were fixed with 4% PFA for 20 min at room temperature and later washed 3 × 5 min in PBS. The cells were permeabilized with Triton X-100 (Sigma Aldrich, Stockholm, Sweden) in PBS (PBS-T 1%) for 1 h and washed 3 × 5 min in PBS. Cells were then incubated in blocking solution in 10% normal donkey serum (NDS) (Millipore, Solna, Sweden)) for 1 h at room temperature and washed 3 × 5 min in PBS. The cells were incubated overnight at 4 °C with primary antibodies: (1:1000) 82E1 (#10323, IBL America, Minneapolis, MN, USA), 6E10 (#803013, BioLegend, London, UK), and amyloid fibrils, OC78 (#ab205341, Abcam, Cambridge, UK). The following day, the cells were washed 3 × 10 min in PBS, incubated with secondary antibodies (donkey anti-mouse or anti-rabbit conjugated to Alexa Fluor 555 or 647 (ThermoFisher Scientific, Gothenburg, Sweden)) for 1 h at room temperature in darkness, washed 3 × 10 min PBS and mounted using ProLong™ Diamond Antifade Mountant (#P36961, Thermo Fisher Scientific, Gothenburg, Sweden). Digital images were obtained using a Nikon confocal A1RHD laser-scanning microscope using a 60× oil-immersion objective. Surface rendering and analysis of the fluorescence signal in confocal z-stacks were performed using Imaris 8.0 software (Bitplane Scientific Software, Belfast, UK). 

### 4.6. Statistical Analysis

The sample size was determined by power analysis in order to detect statistically significant changes within a 25% variation of measured responses. One- and two-way ANOVA followed by Bonferroni-corrected two-group posthoc Student *t*-tests were performed using GraphPad Prism8 (GraphPad Software, San Diego, CA, USA). Experiments were replicated with different amyloid-β-specific antibodies, and 7 to 20 cells were included from two independent experiments.

## Figures and Tables

**Table 1:** Summary of peak positions used in the study.

Wavenumbers (cm^−1^)	Structure
1656 cm^−1^	C=O stretching, Amide I
1550 cm^−1^	C–N stretching; N–H bending, Amide II
1628 cm^−1^	β-sheet (main)
1640 cm^−1^	random coils (unordered)
1656 cm^−1^	α-helix
1691 cm^−1^	β-sheet (weak)
1740 cm^−1^	C=O bond of the ester peak

**Table 2:** Summary of bands ratios used for infrared spectra analysis.

Ratio	Structure
1550 cm^−1^/1656 cm^−1^	protein folding
1628 cm^−1^/1656 cm^−1^	parallel β-sheet features
1640 cm^−1^/1656 cm^−1^	random coils (unordered)
1691 cm^−1^/1628 cm^−1^	anti-parallel β-sheet
1740 cm^−1^/1656 cm^−1^	C=O, lipid oxidation

## Data Availability

The data that support the findings of this study can be provided by the corresponding author upon reasonable request.

## Supplementary Materials

The following are available online at https://www.mdpi.com/article/10.3390/ijms22073430/s1, Figure S1: Cell models to study aggregation of amyloid proteins., Figure S2: Figure S1. Cell models to study aggregation of amyloid proteins. Figure S3: Bright-field images of cells after transfection and FACS sorting. Scale bar is 20 µm. Figure S4: Bright-field images of cells after transfection and FACS sorting. Scale bar is 20 µm. Figure S5: SDS PAGE followed by western blot analysis with specific antibodies against Tau and αSyn ex-pression after transfection. Figure S6: Particle size was quantified by Imaris (Bitplane Scientific Software, Zurich, Switzerland) after surface rendering in confocal images of the cells immunolabeled with antibodies OC and 82E1. Dashed lines indicate a threshold set for the analysis of rendered surfaces in confocal z-stacks that was used for the fluorescent signal quantification.
