# Supplementary material for: Amyloid Structural Changes Studied by Infrared Microspectroscopy in Bigenic Cellular Models of Alzheimer’s Disease
Source: Int J Mol Sci. 2021 Mar 26;22(7):3430. doi: 10.3390/ijms22073430 (PMC8037084; doi:10.3390/ijms22073430)
Supplement: Supplementary file 1 [file ijms-22-03430-s001.pdf]

International Journal of *Molecular Sciences*

MDPI

SUPPLEMENTARY INFORMATION

Amyloid Structural Changes Studied by Infrared Microspectroscopy in Bigenic Cellular Models of Alzheimer's Disease

Agnes Paulus [1,2], Anders Engdahl [1], Yiyi Yang [2], Antonio Boza-Serrano [2], Sara Bachiller [2], Laura Torres-Garcia [3,4], Alexander Svanbergsson [4], Megg G. Garcia [2,3], Gunnar K. Gouras [3], Jia-Yi Li [4], Tomas Deierborg [2,*] and Oxana Klementieva [1,5,*]

[1] Medical Microspectroscopy Laboratory, Department of Experimental Medical Science, Lund University, 22184 Lund, Sweden; agnieszka_agnes.paulus@med.lu.se (A.P.); anders.engdahl@med.lu.se (A.E.)
[2] Experimental Neuroinflammation Laboratory, Department of Experimental Medical Science, Lund University, 22184 Lund, Sweden; yiyi.yang@med.lu.se (Y.Y.); antonio.boza_serrano@med.lu.se (A.B.-S.); sara.bachiller@med.lu.se (S.B.);
[3] Experimental Dementia Research Unit, Department of Experimental Medical Science, Lund University, 22184 Lund, Sweden; megg.garcia@med.lu.se (M.G.G.); laura.torres-garcia@med.lu.se (L.T.-G.); gunnar.gouras@med.lu.se (G.K.G.)
[4] Neural Plasticity and Repair Unit, Department of Experimental Medical Science, Lund University, 22184 Lund, Sweden; alexander.svanbergsson@med.lu.se (A.S.) jia-yi.li@med.lu.se (J.L.)
[5] Lund Institute for Advanced Neutron and X-ray Science (LINXS), 22370 Lund, Sweden
* Correspondence: tomas.deierborg@med.lu.se (T.D.); oxana.klementieva@med.lu.se (O.K.)

Citation: Paulus, A.; Engdahl, A.; Yang, Y.; Boza-Serrano, A.; S. Bachiller; Torres-Garcia, L.; Svanbergsson, A.; Garcia, M.G.; Gouras, G.K.; Li, J.-Y.; et al. Amyloid Structural Changes Studied by Infrared Microspectroscopy in Bigenic Cellular Models of Alzheimer's Disease. *Int. J. Mol. Sci.* **2021**, *22*, x. https://doi.org/10.3390/xxxxx

Academic Editor: Vytautas Smirnovas

Received: 26 February 2021
Accepted: 22 March 2021
Published: 24 March 2021

Publisher's Note: MDPI stays neutral with regard to jurisdictional claims in published maps and institutional affiliations.

Copyright: © 2021 by the authors. Licensee MDPI, Basel, Switzerland. This article is an open access article distributed under the terms and conditions of the Creative Commons Attribution (CC BY) license (http://creativecommons.org/licenses/by/4.0/).

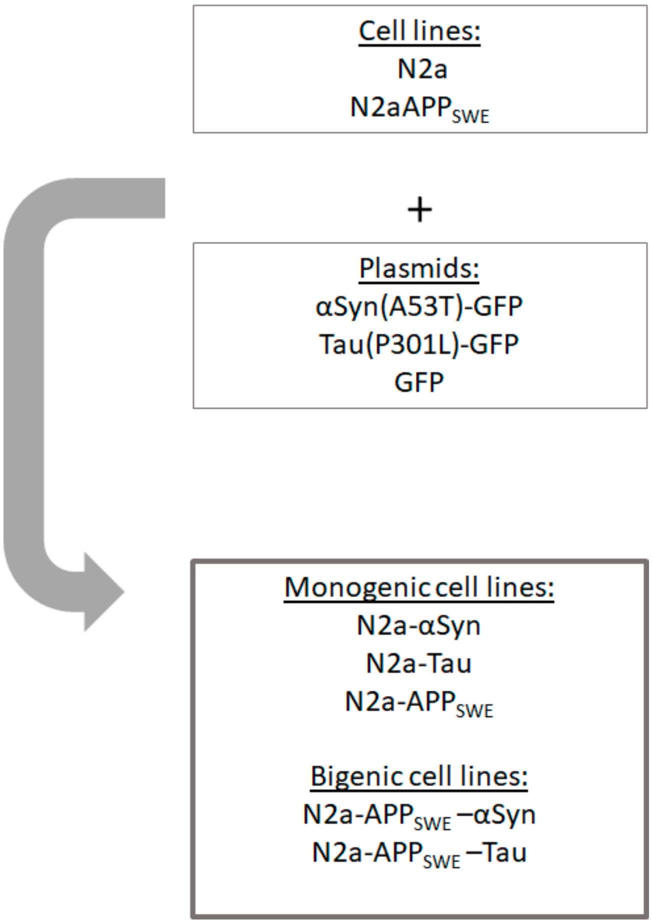

Figure S1. Cell models to study aggregation of amyloid proteins.

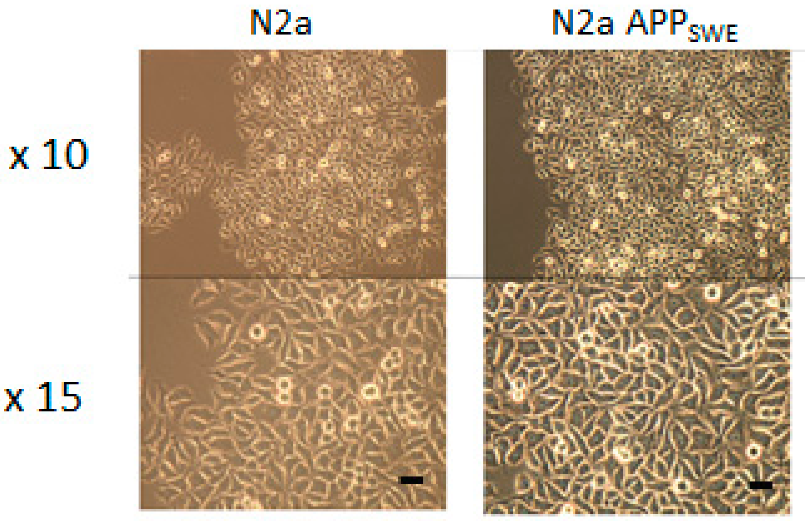

Figure S2. Bright field images of cells before transfection. Scale bar is 20 μm.

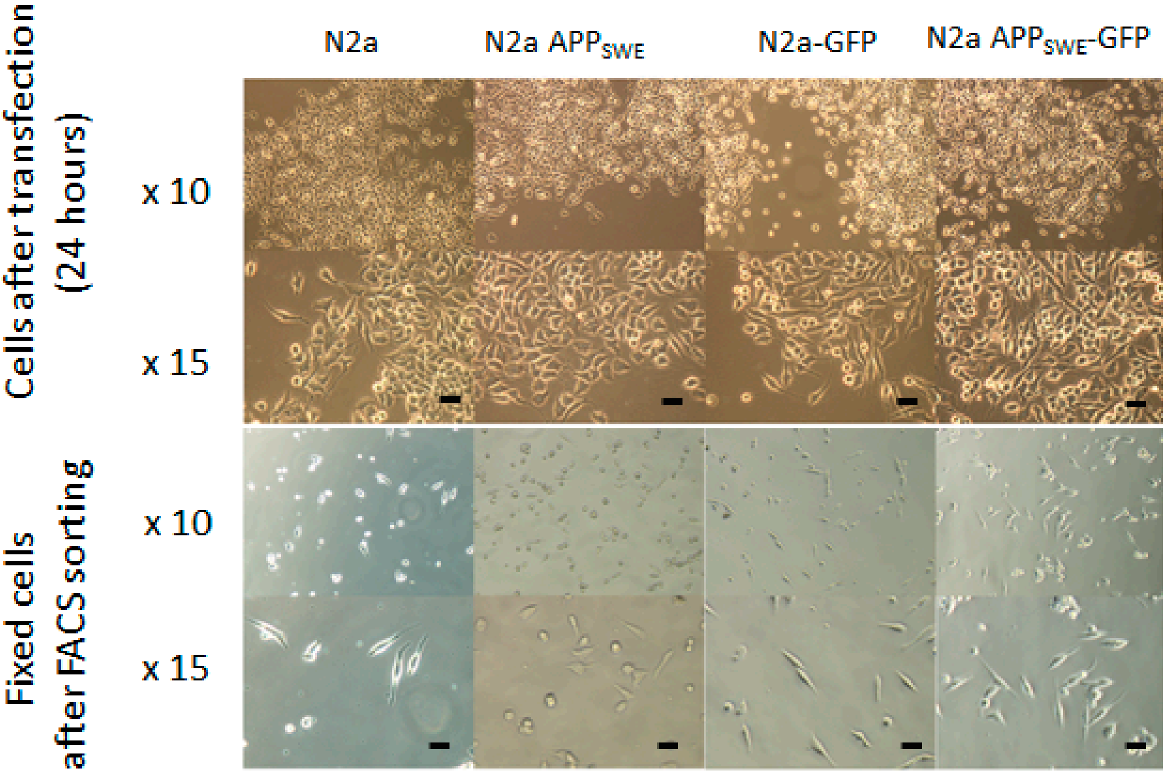

Figure 3. Bright-field images of cells after transfection and FACS sorting. Scale bar is 20 μm.

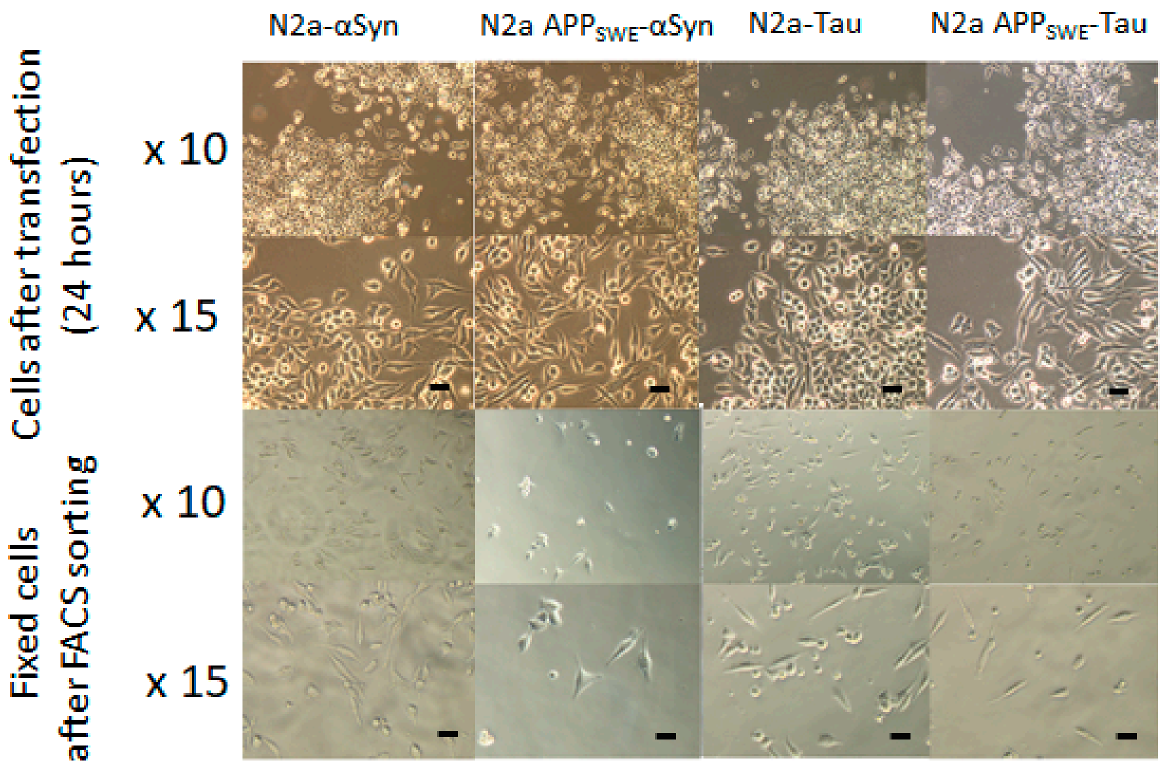

Figure S4. Bright-field images of cells after transfection and FACS sorting. Scale bar is 20 µm.

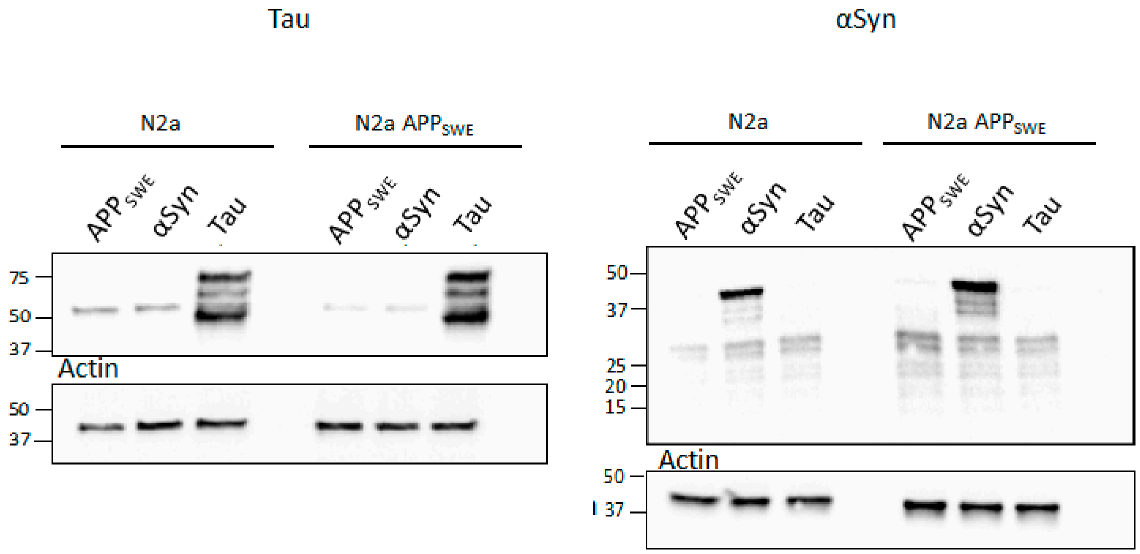

Figure S5. SDS PAGE followed by western blot analysis with specific antibodies against Tau and αSyn expression after transfection.

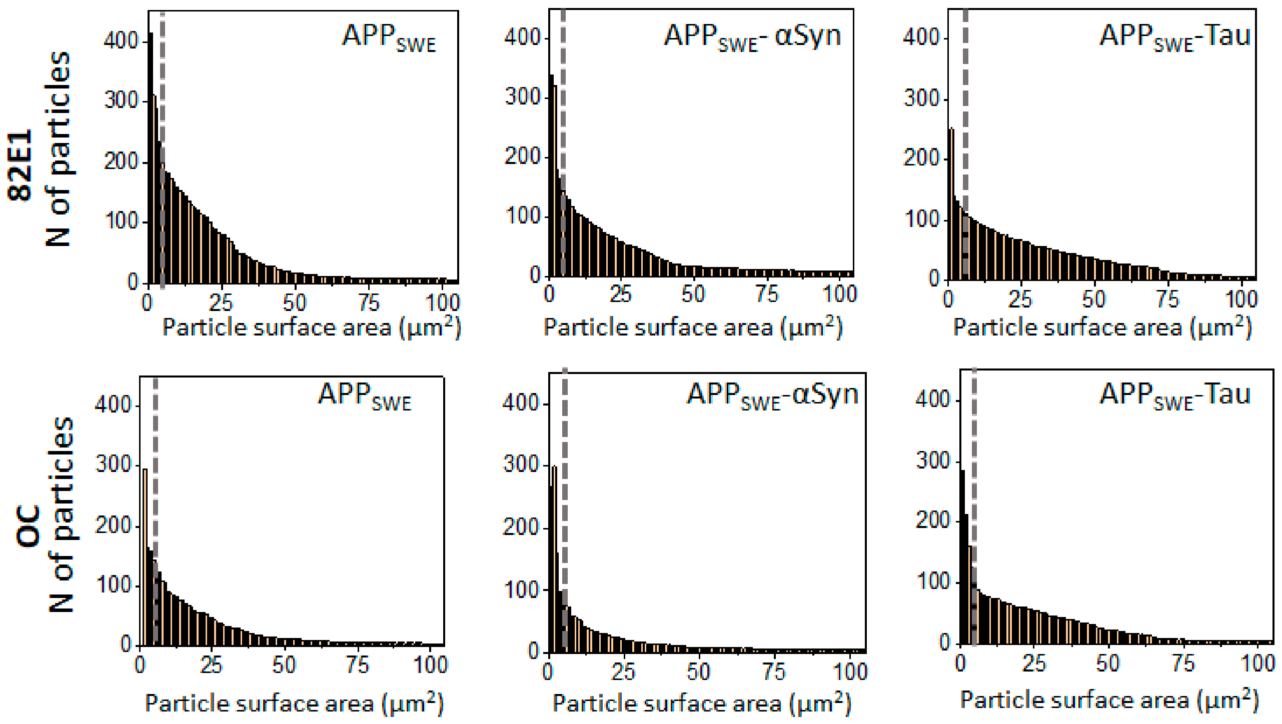

Figure S6. Particle size was quantified by Imaris (Bitplane Scientific Software, Zurich, Switzerland) after surface rendering in confocal images of the cells immunolabeled with antibodies OC and 82E1. Dashed lines indicate a threshold set for the analysis of rendered surfaces in confocal z-stacks that was used for the fluorescent signal quantification.
